# Einsamkeit am Lebensende

**DOI:** 10.1007/s00103-024-03943-0

**Published:** 2024-08-23

**Authors:** Annina Seiler, Sophia Rose Evstigneev, Zehra Hepp, Caroline Hertler, Simon Peng-Keller, David Blum

**Affiliations:** 1 Klinik für Radio-Onkologie, Kompetenzzentrum Palliative Care, Universitätsspital Zürich und Universität Zürich, Rämistrasse 100, 8091 Zürich, Schweiz; 2 Professur für Spiritual Care, Theologische und Religionswissenschaftliche Fakultät, Universität Zürich, Zürich, Schweiz

**Keywords:** Einsamkeit, Lebensende, Identität, Entwicklung, Transzendenz, Loneliness, End of life, Identity, Inner growth, Transcendence

## Abstract

Fortgeschrittene unheilbare Erkrankungen gehen aufgrund des zunehmenden Krankheitsprogresses mit zahlreichen Verlusten und Belastungen einher, welche die Autonomie und Selbstbestimmung sowie das Würdegefühl der Betroffenen erheblich beeinträchtigen und Einsamkeitsgefühle fördern können. Der gesundheitliche Abbau, die zunehmende Symptomlast, der Verlust von sozialen Rollen sowie die Angst vor dem Tod und dem Sterben zählen zu den wichtigsten Risikofaktoren für Einsamkeit am Lebensende. Dieser Artikel bietet einen Überblick über die verschiedenen Dimensionen der Einsamkeit am Lebensende. Die existentielle Einsamkeit wird in Abgrenzung zur emotionalen und sozialen Einsamkeit am Lebensende beleuchtet, Ursachen und gesundheitliche Auswirkungen von Einsamkeit am Lebensende werden diskutiert, auf diagnostische Instrumente wird hingewiesen und Empfehlungen zum Umgang mit der emotionalen, sozialen und existentiellen Einsamkeit am Lebensende werden ausgesprochen. Auch die Einsamkeit pflegender Angehöriger wird thematisiert. Im Artikel weisen wir darauf hin, wie wichtig es ist, der emotionalen und sozialen Einsamkeit am Lebensende frühzeitig entgegenzuwirken. Palliative, psychologische und spirituelle Unterstützung können dabei helfen, zwischenmenschliche Beziehungen zu stärken, Sinn und Bedeutung zu fördern und die negativen Auswirkungen von Einsamkeitsgefühlen auf die Gesundheit und die Lebensqualität zu reduzieren. Im Gegensatz dazu wird die existentielle Einsamkeit als Ausdruck hoher emotionaler Reife betrachtet und kann als entwicklungsfördernde Erfahrung zu einer besseren Verortung des Selbst sowie zur Stärkung von Identität, Würde und Transzendenz am Lebensende beitragen.

## Abstract (en)

Advanced incurable diseases are often accompanied by numerous losses and burdens as the disease progresses, leading to a loss of autonomy, self-determination, and dignity for those affected, all of which can subsequently promote feelings of loneliness at the end of life. Declining health, increasing symptom burden, loss of social roles, and the fear of death and dying are among the key risk factors for loneliness towards the end of life. In this article, we provide an overview of the different dimensions of loneliness experienced at the end of life. We discuss existential loneliness alongside emotional and social loneliness, explore causes and health implications of loneliness at the end of life, suggest diagnostic tools for assessing loneliness, and finally provide recommendations for addressing emotional, social, and existential loneliness at the end of life. The loneliness of caregivers is also discussed. We suggest that addressing social and emotional loneliness early in the course of a terminal illness is crucial. Palliative, psychological, and spiritual support can strengthen interpersonal relationships, foster a sense of meaning and purpose, and alleviate the adverse effects of loneliness on mental and physical health as well as quality of life. In contrast, existential loneliness is considered an expression of profound emotional maturity and can offer opportunities for inner growth, contributing to a more refined sense of self while reinforcing identity, dignity, and transcendence at the end of life.

## Einleitung

„Ich leide an einem angeborenen Herzfehler. Bis vor Kurzem konnte ich ein erfülltes und selbstständiges Leben führen. Leider hat sich meine Gesundheit drastisch verschlechtert, die Einschränkungen nehmen zu, bei der kleinsten Bewegung geht mir der Atem aus, und nachts habe ich Atemnot. Der Kontrollverlust ist für mich sehr schlimm. Ich bin sonst ein aufgestellter und zuversichtlicher Mensch, aber jetzt drückt es mir auf die Stimmung. Ich habe einfach keine Kraft mehr. Ich bin an einen Punkt in meiner Krankheitsgeschichte angekommen, wo der Keller näherkommt. Es ist sehr schwierig, das Leben noch positiv zu sehen, wenn nichts mehr möglich ist. Manchmal denke ich, es wäre mir egal, wenn es vorbei wäre. Ich bin nicht suizidal, das würde ich nie tun. Es ist eher Ausdruck der Verzweiflung. Vielleicht bin ich nicht so leidensfähig, nicht so geduldig. Ich habe die Erwartung, dass noch etwas gehen soll. Mein Kopf ist das Einzige was funktioniert. Manchmal wünschte ich mir, mein Kopf wäre halb so marode wie mein Körper. Das wäre für mich weniger schlimm, für meine Umgebung wahrscheinlich schlimmer. Aber ich bekomme halt alles mit. Der Umgang mit der Ungewissheit ist sehr belastend. Mein Mann meint, ich hätte meine Fröhlichkeit verloren. Es stimmt, ich habe mich verändert, ich habe nicht mehr so viel Freude an den Dingen wie früher. Manchmal versucht er mich zu trösten und meint, ich könne ja noch vieles tun. Aber wenn man in diesem Körper drin ist, kann man nicht viel machen. Und von außen kann man auch nichts machen. Ich muss es alleine aussitzen. Alle sagen, wir verstehen das schon, aber wie es sich wirklich anfühlt, das verstehen nur Betroffene. Mein Leidensdruck ist groß, gleichzeitig möchte ich meinen Mann und meine Familie nicht belasten. Was bringt es ihnen, wenn ich ihnen mitteile, dass ich Angst habe zu ersticken? Sie können mir ja nicht helfen. Ich muss es alleine aushalten.“

Wer eine existentielle Erfahrung an der Grenze oder am Übergang zwischen Leben und Tod macht, tut dies immer allein. Wie das Narrativ dieser Patientin veranschaulicht, kann es sehr schwierig sein, diese Erfahrung selbst mit nahen und vertrauten Menschen zu teilen. Einsamkeit am Lebensende ist ein schwer fassbares Phänomen, weil es ein höchst individuelles Erleben in unterschiedlichen Dimensionen und Ausprägungen darstellt [1]. Menschen mit einer fortgeschrittenen unheilbaren Erkrankung gehören grundsätzlich zu den Risikogruppen für Vereinsamung. Kranke Menschen haben weniger Kraft, ihre sozialen Netzwerke zu pflegen, was in der Folge zu deren Abbau führt. Aufgrund von zunehmender Symptomlast und Kraftverlust, Hospitalisierung und Institutionalisierung können sie weiter marginalisiert werden und die Pflege sozialer Kontakte wird erschwert. Hinzu kommt, das sich schwerkranke Menschen durch die fortschreitende Krankheit und das immer näher rückende Lebensende mit ihrer existentiellen Einsamkeit konfrontiert sehen – die schmerzhafte Erfahrung der eigenen Verletzlichkeit, der individuellen Begrenztheit und sozialen Angewiesenheit [2].

Dieser Artikel bietet einen Überblick über die unterschiedlichen Dimensionen und Folgen der Einsamkeit am Lebensende. Wir werden die existentielle Einsamkeit in Abgrenzung zur emotionalen und sozialen Einsamkeit am Lebensende beleuchten, Ursachen und gesundheitliche Auswirkungen von Einsamkeit am Lebensende diskutieren, auf diagnostische Instrumente hinweisen und Empfehlungen aussprechen, wie Gesundheitsfachpersonen der emotionalen, sozialen und existentiellen Einsamkeit am Lebensende begegnen können.

## Ursachen und gesundheitliche Auswirkungen von Einsamkeit am Lebensende

**Abb. 1:**
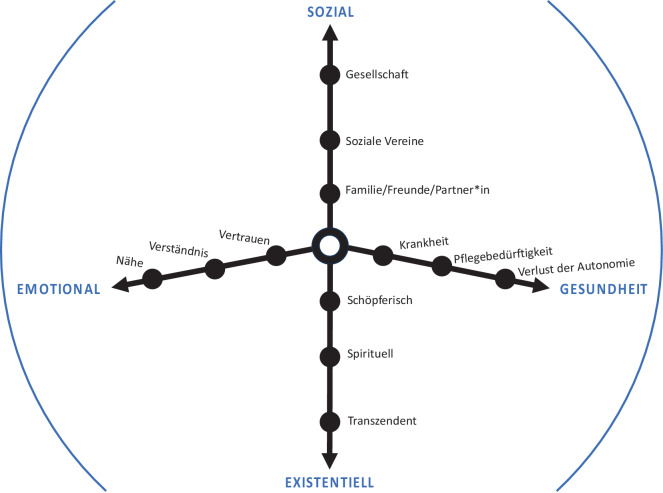
Dimensionen der Einsamkeit bei Menschen mit einer terminalen Erkrankung und ihren pflegenden Angehörigen; adaptiert nach [33]

Fortgeschrittene unheilbare Erkrankungen gehen aufgrund des zunehmenden Krankheitsprogresses mit zahlreichen Verlusten und Belastungen einher, welche die Autonomie und Selbstbestimmung sowie das Würdegefühl erheblich beeinträchtigen und Einsamkeitsgefühle fördern können [3, 4]. Einsamkeit ist eine subjektive Empfindung, die als Diskrepanz zwischen den gewünschten und tatsächlichen sozialen Beziehungen empfunden wird. Sie äußert sich durch ein Gefühl der Leere, der Sehnsucht und einem fehlenden Gefühl der Verbundenheit, wobei Geborgenheit, Liebe oder Wärme vermisst werden [5]. Schwerkranke Menschen sehen sich mit unterschiedlichen Formen der Einsamkeit konfrontiert, wobei insbesondere gegen das Lebensende hin das Erleben von Einsamkeit deutlicher spürbar wird (Abb. 1; [6]).

**Abb. 2:**
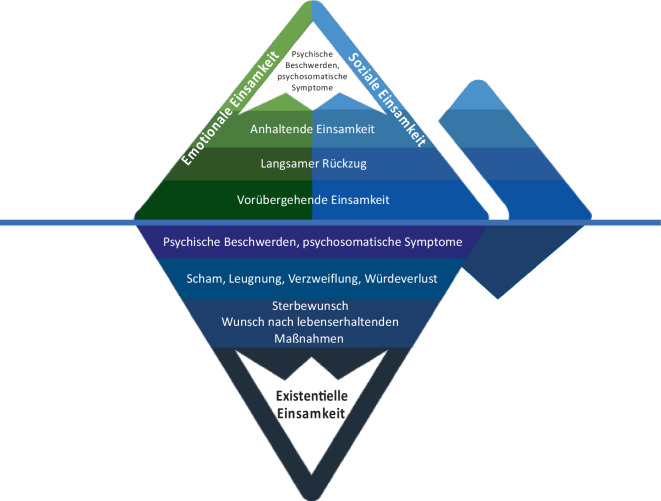
Der Eisberg als Sinnbild der Einsamkeit – zur Veranschaulichung und Unterscheidung zwischen emotionaler, sozialer und existentieller Einsamkeit am Lebensende; adaptiert nach [49]

*Soziale Einsamkeit* entsteht durch fehlende Beziehungen zu Freunden, Familien oder anderen sozialen Netzwerken und kann durch das Alleinsein und die Isolation im Rahmen einer Hospitalisation, Institutionalisierung oder Medikalisierung verstärkt werden, während *emotionale Einsamkeit* das Fehlen vertrauter, verlässlicher, enger oder intimer Bindungen betrifft. Beide Formen der Einsamkeit (Abb. 2: Spitze des Eisbergs) stellen bedeutende Stressoren dar und können tiefgreifende Auswirkungen auf das psychische Wohlbefinden und die körperliche Gesundheit haben [5]. *Existentielle Einsamkeit* beschreibt hingegen das Erleben einer tiefen spirituellen Leere, Hoffnungslosigkeit, Angst und Verzweiflung, die aus dem Bewusstsein von Grenzen, Bindung, Trennung sowie der Sehnsucht nach Sinn und Geborgenheit resultiert – eine Erfahrung, die untrennbar mit dem Menschsein verbunden ist [1].

Am Lebensende ist der Mensch einsam, dahin kann ihm keiner folgen. Ein gutes Sterben verwirklicht sich nach Kübler-Ross, wenn die sterbende Person ihre Beziehungen und das Leben loslassen und einen guten Abschied finden kann [7]. Das Erleben von Einsamkeit und Isolation in der letzten Lebensphase dagegen ist ein ernst zu nehmender gesundheitsschädlicher Risikofaktor, der die Lebensqualität und die verbleibende Lebenszeit beeinträchtigen und die Symptomlast verstärken kann [8]. Einsame Personen am Lebensende nehmen weniger häufig medizinische Behandlungen, dafür häufiger lebenserhaltende Maßnahmen in Anspruch, äußern häufiger den Wunsch nach einem raschen Versterben und versterben tendenziell öfter in einem Alters- und Pflegeheim [6, 9]. Es gibt ebenfalls Hinweise, dass einsame Menschen früher sterben [5]. Einsames Sterben kann in jedem Alter vorkommen. Ein Drittel der palliativen Patienten erlebt Einsamkeit [6], wobei die Einsamkeitsgefühle mit ansteigendem existentiellen Disstress, Verlust der Würde und Krankheitsprogress zunehmen [10, 11].

### Körperliche und psychische Einschränkungen.

Einsamkeit und körperliche sowie psychische Gesundheit beeinflussen sich gegenseitig. Terminale Erkrankungen gehen mit körperlichen Einschränkungen und psychischen Belastungen, u. a. Ängsten, Depressionen oder suizidalen Gedanken, einher [3], was wiederum Einsamkeitsgefühle verstärken kann. Umgekehrt können Einsamkeitsgefühle und Isolation die Entwicklung von körperlichen Beschwerden, Ängsten und depressiven Symptomen begünstigen, wobei Gefühle der Einsamkeit die Symptomlast und das subjektive Schmerzempfinden wesentlich mitbeeinflussen können [12]. Einsamkeit kann ein Risikofaktor für Depression und gleichzeitig ein Symptom der Depression sein [13]. Dies ist besorgniserregend, weil Depression und Einsamkeit bedeutende Risikofaktoren für suizidales Verhalten und Suizid sind [14, 15]. Schutzfaktoren wie sozialer Rückhalt durch Partnerschaft, Familie, Freunde, Arbeitgeber oder soziale Vereine können der Einsamkeit und ihren negativen Folgen immer weniger entgegenwirken. Der gesundheitliche Abfall, die zunehmende Symptomlast, der Verlust von sozialen Rollen sowie die Angst vor dem Tod und dem Sterben zählen zu den wichtigsten Risikofaktoren für Einsamkeit am Lebensende [16, 17]. Diese Risikofaktoren nehmen einerseits mit dem Alter und andererseits mit dem fortschreitenden Krankheitsprogress zu, was dazu führt, dass Betroffene weniger soziale Unterstützung erfahren und das Sterbegeschehen „alleine“ bewältigen müssen [18].

### Schuld und Scham.

Einsamkeit kann auch durch Schuld und Scham sowie ein eingeschränktes Würdeerleben ausgelöst werden, obwohl dieser Zusammenhang in der Literatur weniger untersucht ist, möglicherweise weil diese Gefühle oftmals im Verborgenen wirken. Schuldgefühle können in Bezug auf verschiedene Faktoren entstehen, zum Beispiel durch die Krankheit selbst, das Gefühl, eine Belastung für andere zu sein, oder Angst, bedeutsame Menschen zurückzulassen. Aber auch durch die Lebensbilanzierung können sich Schuldgefühle durch frühere Handlungen, ungelöste Konflikte, unerfüllte Wünsche oder nicht gelebte Lebensaufgaben einstellen. Scham dagegen kann durch krankheitsbedingte körperliche Veränderungen, ein verändertes Körperbild oder Gerüche entstehen, aber auch durch Funktionsverluste wie Bewegungseinschränkungen oder Inkontinenz, Verlust der Autonomie, zunehmende Pflegebedürftigkeit und Abhängigkeit von anderen sowie den damit verbundenen Verlust der Intimität [19]. Kranke und pflegebedürftige Menschen empfinden jede Form von Scham als leidvoll. Sie kann ein Gefühl von Wertlosigkeit auslösen und neben der Einsamkeit depressive Symptome und Sterbewünsche verstärken [20].

### Trauer.

Auch Trauer kann einsam machen. Trauer findet häufig im Stillen statt und ist eine emotionale Reaktion auf die zahlreichen Verluste, die eine schwerkranke Person im Krankheitsverlauf erleben muss – den Verlust der Zukunft, der körperlichen Funktionen, der Kontrolle über das Leben, der Autonomie und Unabhängigkeit und der medizinischen Behandlungsmöglichkeiten [21]. Einsamkeit ist der Trauer sehr ähnlich. Beide Phänomene aktivieren Gehirnregionen, die auch bei körperlich empfundenem Schmerz aktiviert sind [5].

### Wunsch nach raschem Versterben.

Aus der Sterbewunschforschung ist bekannt, dass Einsamkeitsgefühle neben der Belastung durch eine schwere Erkrankung und dem existentiellen Leid den Wunsch nach einem raschen Versterben verstärken können [9, 19]. Schätzungsweise 20 % der palliativen Patienten mit einer Lebenserwartung von weniger als 6 Monate äußern einen Sterbewunsch [9, 13] und Menschen mit einer onkologischen Grunderkrankung haben ein 1,2-fach erhöhtes Suizidrisiko im Vergleich zur gesunden Normalbevölkerung [22]. Insbesondere ältere, multimorbide Patienten sind oft mit der drückenden Belastung der Einsamkeit am Lebensende konfrontiert [10].

### Medikalisierung, Institutionalisierung und Säkularisierung.

Die heutige moderne westliche Kultur bringt weitere Entwicklungen mit sich, die die Einsamkeit am Lebensende begünstigen. Die medizinischen Fortschritte ermöglichen zwar häufig einen späteren Tod, gehen jedoch mit einem belastungsintensiveren, oft multimorbiden Leben und einem längeren und versorgungsintensiven Lebensende einher. Dies reduziert die Möglichkeiten für zwischenmenschliche Beziehungen und erhöht die Belastung durch Einsamkeit. Die Institutionalisierung und Medikalisierung haben das Sterben zwar einfacher und kontrollierbarer gemacht, aber auch dazu geführt, dass das Sterben aus der Verantwortung der Familie und anderer Gemeinschaften gelöst wurde und soziale Bedingungen entstanden sind, die die emotionale Einsamkeit der Sterbenden und ihrer Angehörigen verstärken. Hinzu kommt der Verlust der Sprache und der Sterbe- und Trauerkultur, was den Umgang mit dem Lebensende, Tod und Trauer erschwert. Schließlich behindert die zunehmende Säkularisierung die Auseinandersetzung mit der eigenen Endlichkeit und Vergänglichkeit und trägt somit zu einem Grundgefühl der existentiellen Einsamkeit bei [23].

## Existentielle Einsamkeit

Die Diagnose einer schweren unheilbaren Erkrankung sowie die Konfrontation mit dem Lebensende können das Selbstverständnis, die eigene Identität und bis dahin gelebte Annahmen über sich selbst, die Beziehungen und die Welt grundlegend infrage stellen [24]. Die existentielle Einsamkeit beschreibt nicht das Fehlen von sozialen Beziehungen, sondern ein Gefühl der „Entfremdung von sich selbst und der Umwelt“ und die Erfahrung, dass wir in existentiellen Grenzsituationen, wie die Geburt oder der Tod, auf uns alleine gestellt sind [25]. Diese Einsamkeitserfahrung ist an die *Conditio humana *– die dem Menschen eigene Grundverfassung – gebunden und bringt die Tatsache unserer Verletzlichkeit, unserer sozialen Angewiesenheit und unserer Endlichkeit und Vergänglichkeit zum Ausdruck. Sie reflektiert die Urangst vor dem Alleinsein [18]. Existentielle Einsamkeit ist besonders angesichts der eigenen Sterblichkeit erfahrbar, kann aber auch bei schweren psychischen Krisen, chronischen Erkrankungen oder nach einem schweren Verlust erlebt werden [25]. Existentielle Einsamkeit ist in allen Facetten des menschlichen Erlebens eine bedrohliche Erfahrung, in der kein Sinn und keine Bedeutung mehr spürbar sind und die trotz höchst befriedigender Verbindungen zu anderen Menschen erlebt werden kann. Diese Einsamkeitserfahrung ist geprägt von tiefem Leid, spiritueller Leere, Traurigkeit, Kontrollverlust, Ängsten, Verzweiflung und Hoffnungslosigkeit [18]. Der Verlust der körperlichen und seelischen Integrität, der Verlust von Sinn und Bedeutung im Leben, der Verlust der eigenen Autonomie und Selbstständigkeit, Schuldgefühle, Reue, der schmerzhafte Abschied von geliebten Menschen und das Bewusstsein, dass auch am Ende eines erfüllten Lebens Unerledigtes zurückbleibt, können Gefühle der existentiellen Einsamkeit verstärken und einen hohen Leidensdruck verursachen. Umgekehrt kann das Erleben der existentiellen Einsamkeit Schmerzen, Ängste oder Unruhe intensivieren [12]. Die Erfahrung der *existentiellen Einsamkeit *ist wohl die komplexeste Form der Einsamkeit, die in einer existentiellen Tiefe gründet und schwer erfassbar ist (Abb. 2: Teil des Eisbergs der unter Wasser liegt und nicht sichtbar ist). In diesem Artikel verfolgen wir die Hypothese, dass die existentielle Einsamkeit ein wesentliches Moment der präterminalen Einsamkeit darstellt. Gelingt es jedoch, die existentielle Einsamkeit zu überwinden, kann dies zu persönlichem Wachstum, innerem Frieden, Akzeptanz, Wiederbegegnung und Versöhnung führen.

### Bedeutung und Chancen.

Aus philosophischer Sicht stellt die existentielle Einsamkeit kein Gesundheitsproblem dar, sondern ist Ausdruck unseres Bedürfnisses nach Sinn und Bedeutung im Leben, nach Spiritualität und Transzendenz und wird als wesentlicher Bestandteil der menschlichen Existenz gesehen [25, 26]. Diese Form der Einsamkeit fordert schwerkranke Menschen heraus, sich selbst zu begegnen und sich den tiefgründigen Fragen nach der eigenen Existenz, dem Sinn und der Bedeutung des Daseins und des Lebens zu stellen [27]. In der existentiellen Einsamkeitserfahrung spiegeln sich die Sehnsüchte, Leiden und unerfüllte Wünsche jedes Einzelnen wider sowie der Wunsch nach Trost, Liebe, Zuwendung und Fürsorge [2]. Interessanterweise reflektiert die existentielle Einsamkeit gleichzeitig die Kehrseite unseres Strebens nach Autonomie und Selbstverwirklichung, dessen Konsequenzen von einigen erst am Lebensende in radikaler Beziehungslosigkeit und Einsamkeit erlebt werden [26].

Die existentielle Einsamkeit hat aber nicht nur einen leidvollen Charakter, sondern kann auch eine sinnvolle, kreative, wohltuende und entwicklungsfördernde Kraft haben. Die Erfahrung der existentiellen Einsamkeit kann die Selbstwahrnehmung steigern und zu einer konstruktiven Auseinandersetzung mit sich selbst führen. Einsamkeit kann der Ort sein, in dem das Individuum seine fragile Existenz und Echtheit als Einzelwesen erfährt [28]. Aus theologischer und philosophischer Perspektive birgt die existentielle Einsamkeit eine schöpferische und spirituelle Ressource, die eine höhere Bewusstseinsstufe, einen stärkeren Glauben, eine transzendente Erfahrung sowie Sinn- und Bedeutungsfindung erst möglich macht [29]. Transzendenz am Lebensende bezieht sich auf die Erfahrung, die über die physischen und emotionalen Begrenzungen der gegenwärtigen Realität hinausgeht und als ein Gefühl von Frieden, Akzeptanz und spiritueller Erfüllung wirkt. Kann also die existentielle Einsamkeit als menschliche Erfahrung ausgehalten, akzeptiert und in die eigene Lebensgeschichte integriert werden, öffnen sich Wege zu persönlicher Entwicklung, moralischer Sensibilität, Stärkung der Beziehungen, spirituellem Bewusstsein oder transzendenter Bedeutungsfindung [16]. Transzendenzerfahrungen am Lebensende können Sterbenden und Angehörigen helfen, dem Tod mit Würde und innerem Frieden zu begegnen, was zu einer bedeutsameren, hoffnungsstärkenden, tröstenden und weniger von Angst geprägten Sterbeerfahrung beiträgt [30]. Die Befähigung zur existentiellen Einsamkeit ist in diesem Sinne Ausdruck höchster emotionaler Reife [28].

## Einsamkeit pflegender Angehöriger

Einsamkeit betrifft nicht nur kranke und pflegebedürftige Menschen, sondern auch ihre pflegenden Angehörigen. Die Sorge um den kranken nahestehenden Menschen mündet für viele pflegende Angehörige in Einsamkeit. Pflegende Angehörige sind für eine hochwertige Versorgung kranker und pflegebedürftiger Menschen unverzichtbar. Es ist pflegenden Angehörigen zu verdanken, dass schwerkranke Personen so lange wie möglich zu Hause leben können. Pflegende Angehörige sind eine wichtige Säule der Gesundheitsversorgung und entlasten die öffentliche Hand enorm. Mit dem demografischen Wandel und der zunehmenden Lebenserwartung nehmen nicht nur die Komplexität der Pflegebedürfnisse zu, sondern auch die Anforderungen an pflegende Angehörige, die häufig zwischen beruflicher Tätigkeit, Familie und Pflege von kranken Angehörigen jonglieren müssen [31].

Die Belastung von pflegenden Angehörigen kann sehr hoch sein und diese an ihre emotionalen und körperlichen Grenzen bringen. Mit fortschreitendem Krankheitsprogress und zunehmender Pflegebedürftigkeit verzichten pflegende Angehörige häufig auf ihre Freizeit und sinnstiftenden Aktivitäten und vernachlässigen ihre sozialen Kontakte. Diese Ressourcenanpassung dient der Sicherung der häuslichen Betreuung und Pflege, erhöht jedoch auch das Risiko der Einsamkeit und der Entwicklung von gesundheitlichen Beschwerden [32]. Fehlende soziale Kontakte können das Gefühle der Erschöpfung und Überlastung nicht mehr kompensieren.

Aus einer anfänglichen emotionalen oder sozialen Einsamkeit entwickelt sich allmählich eine existentielle Einsamkeit, die das Lebensgefühl durchdringt. Rollenveränderungen, Entfremdung, Überforderung, Schuld, innere Zerrissenheit, schlechtes Gewissen, Verzicht auf eigene Bedürfnisse, Gefühle der Hilf- und Nutzlosigkeit, Verlust der Intimität in der Partnerschaft sowie eingeschränkte Möglichkeiten, das Leben zu planen, zu gestalten oder Ziele zu verfolgen, können in eine existentielle Erfahrung von Einsamkeit münden [33].

Die antizipatorische Trauer – die Wahrnehmung des drohenden Verlusts – verstärkt Einsamkeitsgefühle. Der tatsächliche Verlust und die einsetzende Trauer schließlich können die Identität, Geborgenheit und Vertrautheit des pflegenden Angehörigen gefährden und eine existentielle Sinnkrise auslösen. Insbesondere bei Personen, die unter anhaltender Trauer leiden, besteht ein erhöhtes Risiko, einsam zu werden [34].

Bei der Begleitung und Unterstützung von palliativen Patienten sind Gesundheitsfachpersonen ebenfalls dazu angehalten, auf die Bedürfnisse von pflegenden Angehörigen zu achten und diese dabei zu unterstützen, die Einsamkeit zu überwinden, Stress zu reduzieren und frühzeitige professionelle Hilfe zur Trauerverarbeitung in Anspruch zu nehmen.

## Psychometrische Instrumente zur Erfassung von Einsamkeit am Lebensende

Die deutsche S3-Leitlinie für Palliativmedizin empfiehlt die regelmäßige Erfassung und Einschätzung psychischer Belastungen bei Patienten mit einer unheilbaren Krebserkrankung [35]. Dabei ist auch die Erfragung der Einsamkeit wichtig, weil Patienten diese selten von sich aus berichten. Eines der häufigsten und etabliertesten Instrumente zur Erfassung der emotionalen und sozialen Einsamkeit ist die *UCLA Loneliness Scale*, welche in unterschiedliche Sprachen übersetzt und validiert wurde [36]. Instrumente zur Erfassung der *existentiellen Einsamkeit* gibt es nur wenige. In diesem Zusammenhang nennbare psychometrische Instrumente sind der *Existential Loneliness Questionnaire *von Mayers et al. [37] oder *die Existential Loneliness Scale *von Pinel et al. [38]. Beide Instrumente sind unseres Wissens jedoch nur in der englischen Sprache validiert.

## Unterstützungsmöglichkeiten und therapeutische Interventionen

Es ist wichtig, der emotionalen und sozialen Einsamkeit am Lebensende frühzeitig entgegenzuwirken. Dafür bedarf es der Unterstützung und Eingebundenheit in unterschiedliche soziale Netzwerke. Der Einbezug von nahen Familienangehörigen, aber auch die Unterstützung durch Nachbarschaftshelfer, Pflegeheime oder die ambulante und stationäre Palliativ- und Hospizversorgung können die Integration von schwerkranken Menschen verbessern und Einsamkeitsgefühlen vorbeugen [17]. Auch am Lebensende kann der Mensch weiterhin am Leben teilnehmen und etwas erleben: sei es durch Alltägliches wie Freuden, Enttäuschungen, Lachen oder Streitigkeiten, Gesten oder Berührungen. Die Unterstützung durch *Palliative Care* und *Spiritual Care* sowie die Aktivierung durch Kunst‑, Musik- oder Ergotherapie können helfen, Inhalte, Bereiche, Tätigkeiten oder Aufgaben zu finden, die den Betroffenen Freude bereiten und trotz der Belastungen durch die fortschreitende Erkrankung und die zunehmenden Verluste Sinn und Bedeutung vermitteln.

Im Gegensatz zur emotionalen und sozialen Einsamkeit wird die existentielle Einsamkeit weniger als Gesundheitsproblem gesehen, sondern vielmehr als eine herausfordernde Erfahrung, die jeder Mensch im Rahmen seiner Entwicklung durchläuft. In der Auseinandersetzung mit der existentiellen Einsamkeit liegt die Chance zu einer besseren Verortung des Selbst und zur Stärkung von Autonomie, Würde und Transzendenz des Menschen am Lebensende. Vielen kranken Menschen ist diese existentielle Auseinandersetzung aus eigener Kraft jedoch nicht möglich. Umso wichtiger ist die Schulung von Gesundheitsfachpersonen hinsichtlich der Bedeutung der existentiellen Einsamkeit für kranke und sterbende Menschen, damit diese wahrgenommen und auch hinter der Schamgrenze behutsam angesprochen werden kann [1]. Ein Leitfaden zur Begegnung der Einsamkeit am Lebensende ist in der Infobox dargestellt.

Menschen am Lebensende benötigen vor allem seelischen Beistand, Fürsorge und Zuwendung von nahen unterstützenden Personen, welche die Konfrontation mit dem Leid sowie der eigenen Endlichkeit und Vergänglichkeit aushalten und ihnen Hoffnung und Kraft vermitteln können. Die Stärkung der Hoffnung spielt im Umgang mit existentieller Einsamkeit am Lebensende eine bedeutende Rolle. Hoffnungslosigkeit geht häufig mit Sterbewünschen einher, während Hoffnung als Schutzfaktor vor Stress und psychischen Belastungen wirkt [39]. Die Bedeutung der Hoffnung am Lebensende ist gut untersucht. Hoffnung steht als Gegenbegriff zu Verzweiflung und hilft Menschen ihr Leid auszuhalten und dem Leben trotz Einschränkungen und Verlusten Sinn und Bedeutung zu verleihen [15]. Hoffnung verkörpert einen starken Handlungsantrieb, richtet Menschen in ihrem Leid auf und stärkt ihre Willenskraft. Über die Hoffnung erschließen sich Menschen neue Existenz- und Handlungsmöglichkeiten und bewahren dadurch ihre Handlungsfähigkeit, Autonomie und Selbstbestimmung, die häufig über den Tod hinauswirken [40]. Die Haltung von Gesundheitsfachpersonen gegenüber der Hoffnung von Patienten oder Angehörigen ist ebenfalls eine wichtige Ressource im Umgang mit existentieller Einsamkeit [39].

Menschen mit einer schweren fortgeschrittenen Erkrankung und ihre Angehörigen sind manchmal wie fallende Blätter und müssen aufgefangen werden. Ein bisschen so, als würde man ein Kind auf die Arme nehmen, um es zu trösten, genauso bedürfen Menschen im existentiellen Disstress aufgrund der infausten Prognose und des nahenden Lebensendes oder eines drohenden Verlusts einer freundschaftlichen Umarmung. Die palliative Fürsorge und Zuwendung versucht nun durch „palliare“ (lat. Ummantelung), eine Haltung, die wohl am besten durch „*Ich halte Sie*“ übersetzt wird, Menschen (Patienten und Angehörige) in ihrem existentiellen Leid aufzufangen. Palliative Care und Spiritual Care können die existentielle Einsamkeit lindern, indem ein sicherer Raum für bewusste zwischenmenschliche Begegnungen geschaffen wird und Patienten und ihre Angehörigen unterstützt werden, sich mit dem Leben und dem Lebensende auseinanderzusetzen.

Die Thematisierung von Lebensendthemen mit der Familie fällt vielen Betroffenen schwer. Auch Angehörige fühlen sich häufig mit der Lebensendsituation überfordert. Es kann schwierig sein, die richtigen Worte zu finden und Ängste, Gefühle, Wünsche oder Bedürfnisse auszudrücken. Der Leitfaden zur „Würdezentrierten Therapie“ (Dignity Therapy) ermutigt Patienten und ihre Angehörigen ein Gespräch über das Lebensende zu führen und Fragen zu formulieren, die für die Familie wichtig und bedeutungsvoll sind [41]. Die Dignity Therapy ist ein hilfreiches Instrument zur Würdigung der bedeutungsvollen Momente und Erinnerungen im Leben und schafft gleichzeitig ein einzigartiges Vermächtnis für Angehörige. Durch Erzählen von Erinnerungen, Wünschen und Anliegen der Patienten soll die Wertschätzung für das eigene Leben erhöht, die Sinnfindung unterstützt und die Bedeutung der eigenen Lebensgeschichte erkannt werden [4]. Das Erzählen der eigenen Lebensgeschichte als Prozess der Sinnstiftung hat dabei eine wichtige Bewältigungsfunktion [42]. Die erzählerische Aufarbeitung der Krankheitserfahrung – der Einbruch durch die Erkrankung, das veränderte Selbstverständnis, Erfahrungen von Verlusten, Schwäche und Schmerzen, aber auch Erfahrungen mit Menschen und Institutionen – kann helfen, Schmerzen zum Ausdruck zu bringen, Verluste zu betrauern und Trost zu finden [43]. Die Selbsterzählung unterstützt die Auseinandersetzung mit Lebensrupturen sowie der Endlichkeit und Vergänglichkeit und kann zur psychischen Entwicklung und zu persönlichem Wachstum beitragen, Ängste lindern und das eigene Leben bereichern [44]. Dadurch lässt sich auch eine andere Perspektive auf das Sterben finden, was das Erleben von existentieller Einsamkeit mildern und unterschiedliche Formen der Selbsttranszendenz fördern kann [45].

Schließlich möchten wir in diesem Artikel die Belastung von Gesundheitsfachpersonen nicht unerwähnt lassen. Gesundheitsfachpersonen sind in der Begleitung und Betreuung von palliativen Patienten und ihren Angehörigen stets angehalten, sich empathisch gegenüber den Ängsten, Nöten und Sorgen der Patienten zu öffnen. Dies kann jedoch Einsamkeit und moralischen Disstress hervorrufen und das Risiko für die Entwicklung von stressbezogenen Krankheiten wie Burnout oder Depression erhöhen [46]. Wir möchten die Wichtigkeit der Selbstfürsorge in der Begleitung und Unterstützung von schwerkranken Menschen und ihren Angehörigen betonen. Supervision, Balintgruppen sowie Ansätze aus der narrativen Medizin unterstützen die Selbstreflexion und können helfen, die Selbstfürsorge zu stärken und Belastungen von Gesundheitsfachpersonen zu reduzieren [47].

## Fazit

Psychische und körperliche Belastungen am Lebensende gehen häufig mit Einsamkeitserfahrungen einher. In der Begleitung und Betreuung von palliativen Patienten und ihren Angehörigen ist es wichtig, die verschiedenen Dimensionen der Einsamkeit zu erkennen und zu wissen, wie man diesen Leidenserfahrungen begegnen und sie lindern kann. Die Stärkung der sozialen Eingebundenheit, die Förderung der Kommunikation sowie die Unterstützung von Bedeutungsfindung und Aufrechterhaltung der Hoffnung am Lebensende durch palliative, psychologische und spirituelle Begleitung und Unterstützung können helfen, Einsamkeitsgefühle am Lebensende zu mildern und die negativen Auswirkungen auf die Gesundheit und die Lebensqualität zu reduzieren. Die Aufgabe von Palliative Care und Spiritual Care besteht folglich nicht nur in der Linderung der Einsamkeit, sondern auch in der Stärkung der zwischenmenschlichen Beziehungen und der Förderung des persönlichen und spirituellen Wachstums, das aus dem Erleben der existentiellen Einsamkeit entstehen kann.

### Infobox Leitfragen zur Begegnung der Einsamkeit am Lebensende; adaptiert nach [48]

- Wie entstand die Einsamkeit – wie fing es an?
- Wie zeigt sie sich – in Äußerungen, im Erleben, in Bildern oder Symbolen, in Texten, in der Begegnung mit Menschen?
- Wie fühlt sie sich an?
- Wie klingt sie – als Musik, als Stimme, als Geräusch?
- Was fehlt Ihnen?
- Welche Impulse können hilfreich sein? (Gespräche; kunsttherapeutische Methoden; Ergotherapie; Zeit mit Menschen, die von Bedeutung sind; Natur; Tiere)
- Welche Wege führen hinaus?
- Wo zeigen sich Ressourcen, wo finden sich Lösungen?
